# P INK1/PARKIN mitophagy: a potential regulator of mitochondrial homeostasis in functional dyspepsia

**DOI:** 10.3389/fphys.2026.1881965

**Published:** 2026-09-15

**Authors:** Peizhe Li, Yu Wang, Yanan Dong, Xin Zhang, Kun Shang

**Affiliations:** 1 Department of Acupuncture and Massage, Changchun University of Chinese Medicine, Changchun, Jilin, China; 2 Department of Basic Medical Sciences, Changchun University of Chinese Medicine, Changchun, Jilin, China

**Keywords:** functional dyspepsia, gastrointestinal motility, mitophagy, oxidative stress, PINK1/PARKIN signaling pathway

## Abstract

Functional dyspepsia (FD) is a highly prevalent functional gastrointestinal disorder with limited long-term treatment efficacy and high recurrence rates, while its upstream pathogenic mechanisms remain incompletely defined. Mitochondrial dysfunction and defective mitophagy represent key pathological events underlying FD pathogenesis. Beyond the PINK1/PARKIN-dependent ubiquitinated mitophagy cascade, the non-ubiquitin BNIP3/NIX mitophagy axis also mediates gastrointestinal mitochondrial quality control under hypoxic and inflammatory stimuli. As the canonical signaling pathway governing mitophagy and mitochondrial homeostasis, the PINK1/PARKIN axis potentially participates in regulating gastrointestinal energy metabolism, restraining oxidative stress, modulating visceral sensation, and maintaining mucosal barrier integrity. This narrative review elaborates the molecular regulatory mechanisms of the PINK1/PARKIN pathway and proposes a tentative pathological cascade: impaired PINK1/PARKIN signaling – defective mitophagy – gastrointestinal dysmotility, visceral hypersensitivity and chronic low-grade inflammation – FD onset and progression. We further summarize prominent unresolved research gaps in this field. Further investigations targeting this pathway may offer novel theoretical foundations and promising intervention targets for precision diagnosis and management of FD.

## 1 Introduction

Functional dyspepsia (FD) is a highly prevalent functional gastrointestinal disorder worldwide, with an overall population prevalence ranging from 15% to 25%. Approximately 18.9% of Chinese adults suffer from FD, and its annual recurrence rate exceeds 70%. This disease imposes substantial socioeconomic and clinical burdens on healthcare systems and severely impairs patients’ quality of life (Ford et al., 2020; Wang and Liu, 2026). Diagnosed according to Rome IV criteria, FD is characterized by persistent upper abdominal discomfort without detectable organic lesions such as mucosal erosion, ulcers or malignant neoplasms. Current therapeutic regimens mainly rely on prokinetic agents, acid suppressants and visceral hypersensitivity modulators, yet these conventional interventions exhibit limited long-term treatment efficacy, and symptom relapse is very common (Vanheel et al., 2017). Existing pathophysiological studies of FD have primarily focused on gastrointestinal hypomotility, visceral hypersensitivity, brain-gut axis imbalance, intestinal microbiota dysbiosis, low-grade mucosal inflammation and psychological comorbidities (Mimidis and Tack, 2008). Nevertheless, most of these works only describe downstream phenotypic changes, lacking comprehensive exploration of core upstream signaling pathways that maintain cellular and mitochondrial homeostasis.

In recent years, accumulating evidence has highlighted the vital contribution of mitochondrial dysfunction to FD development. Gastric mucosal samples from FD patients display reduced mitochondrial membrane potential, elevated mtDNA deletion frequency, markedly increased oxidative stress markers (including ROS and MDA), alongside impaired ATP synthesis within gastric smooth muscle cells. These observations indicate mitochondrial damage and defective clearance serve as pivotal drivers of FD pathological progression (Liu et al., 2025). Mitophagy acts as the core homeostatic mechanism controlling mitochondrial quantity and quality; it selectively eliminates injured mitochondria to block ROS leakage, energy depletion and subsequent cellular apoptosis (Li et al., 2023). Mammalian gastrointestinal cells possess two independent mitophagy regulatory systems: the ubiquitin-dependent PINK1/PARKIN cascade, and the non-ubiquitin receptor-mediated BNIP3/NIX axis. BNIP3 and NIX are outer mitochondrial membrane mitophagy receptors activated by hypoxia, oxidative stress and chronic inflammation; they directly bind LC3 independent of PARKIN-mediated ubiquitination to initiate mitophagy. In gastrointestinal epithelial and enterochromaffin cells, BNIP3/NIX expression is upregulated under the chronic hypoxic mucosal microenvironment of FD, partially compensating for insufficient PINK1/PARKIN-dependent mitophagy to clear damaged organelles. Current FD research mainly centers on the PINK1/PARKIN pathway, whereas the regulatory roles and pathological contributions of the BNIP3/NIX axis in gastric mucosal mitochondrial homeostasis remain poorly characterized, representing an underexplored research direction worthy of further exploration (Garza-Lombó et al., 2020; Sulkshane et al., 2021). The PINK1/PARKIN cascade is the most extensively characterized mitophagy signaling module, and preclinical studies have confirmed its essential regulatory functions in neurodegenerative diseases, malignant tumors, myocardial ischemia and inflammatory bowel disorders (Narendra and Youle, 2024).

By integrating cross-disciplinary preclinical and clinical evidence, this narrative review elaborates the molecular regulatory mechanisms of the PINK1/PARKIN pathway, constructs tentative logical connections between this pathway and core pathological phenotypes of FD, identifies existing research limitations, and proposes feasible follow-up research strategies. This work aims to provide new perspectives for mechanistic research and precise therapeutic development of FD.

## 2 Molecular regulatory mechanism and mitochondrial protective effects of the PINK1/PARKIN pathway

### 2.1 Activation and molecular regulation of the PINK1/PARKIN pathway

PINK1 (PTEN-induced putative kinase 1) is an evolutionarily conserved serine/threonine protein kinase, whereas PARKIN is an E3 ubiquitin ligase belonging to the RING finger family. These two molecules act in concert to recognize, tag, and degrade damaged mitochondria (Iguchi et al., 2013), thereby constituting a core signaling axis that mediates selective mitophagy and is indispensable for preserving mitochondrial network integrity, supporting cellular energy metabolism, and maintaining cell viability (Narendra et al., 2010). In healthy mitochondria with intact membrane potential and structural integrity, the PINK1/PARKIN pathway remains in a quiescent state. Newly synthesized PINK1 precursor proteins in the cytosol are translocated into the mitochondrial inner membrane via the collaborative actions of the translocase of the outer mitochondrial membrane (TOM complex) and the translocase of the inner mitochondrial membrane (TIM complex). Subsequently, PINK1 is specifically cleaved and degraded by the inner membrane protease PARL, followed by further elimination through the ubiquitin–proteasome system. Consequently, PINK1 is maintained at an extremely low basal level in both the cytosol and mitochondria, failing to trigger downstream signaling cascades, and the entire pathway remains silent under physiological conditions (Nguyen et al., 2016). (**Figure 1**).

**Figure 1:**
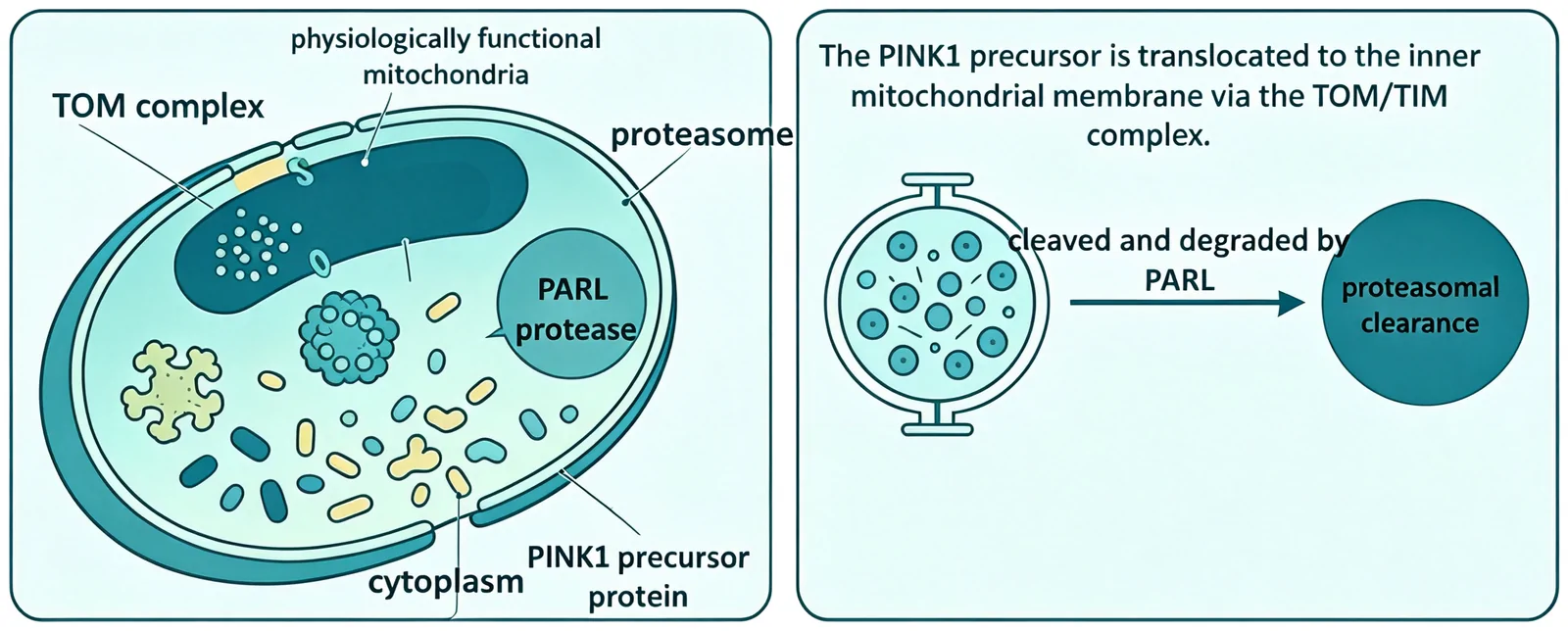
PINK1PARKIN-mediated mitochondrialautophagy.

Mitochondrial injury and membrane potential depolarization can be triggered by oxidative stress, metabolic disturbance and other harmful stimuli. These stresses block the transmembrane transport machinery of mitochondria. The impaired transmembrane trafficking prevents PINK1 from entering the mitochondrial inner membrane, leading to its accumulation and dimerization on the outer mitochondrial membrane, which triggers PINK1 autophosphorylation and subsequent kinase activation (Wang et al., 2025). Activated PINK1 specifically phosphorylates ubiquitin at the S65 site to generate high-affinity phosphorylated ubiquitin chains. This molecular signal efficiently recruits autoinhibited PARKIN from the cytosol, driving its translocation and anchoring to the outer mitochondrial membrane, accompanied by the relief of its conformational inhibition and full activation of E3 ubiquitin ligase activity (Eiyama and Okamoto, 2015). Furthermore, PINK1 directly phosphorylates the conserved homologous sites of PARKIN, thereby amplifying catalytic efficiency and establishing a positive regulatory feedback loop. (**Figure 2**).

**Figure 2:**
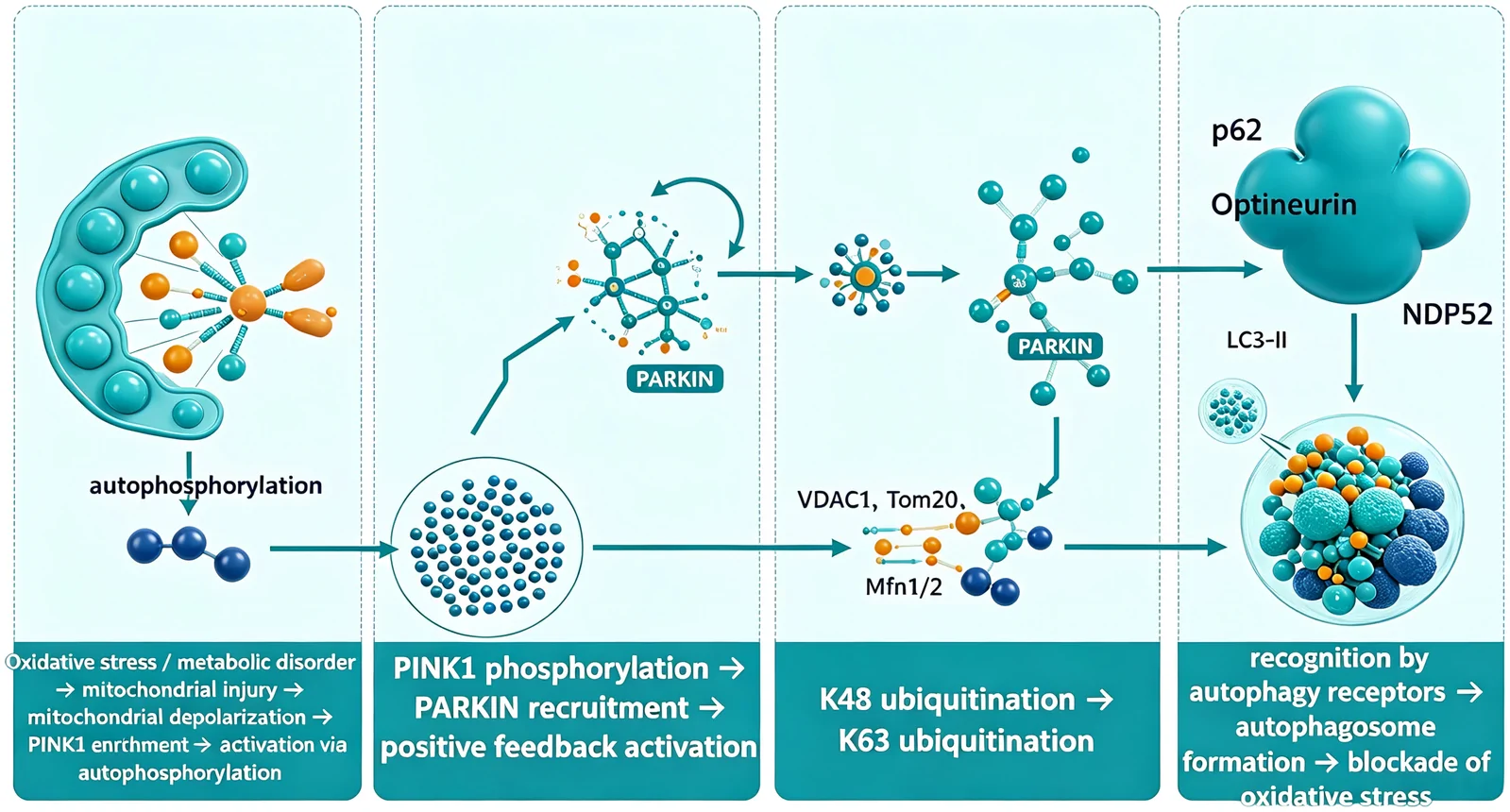
Process of damaged mitochordria via PINK1PARKIN-mediated mitophagy

Once fully activated, PARKIN catalyzes K48- and K63-linked polyubiquitination of multiple outer mitochondrial membrane substrates, including voltage-dependent anion channel 1 (VDAC1), translocase subunit Tom20, and mitochondrial fusion proteins Mfn1/2. Specifically, K48-linked polyubiquitination targets substrate proteins for proteasomal degradation, while K63-linked polyubiquitination acts as a canonical targeting signal for selective autophagy receptors, such as p62, NDP52, and optineurin. These receptors further bind to LC3-II, a core autophagy-related protein, thereby facilitating autophagic membrane extension and the encapsulation of damaged mitochondria to form autophagosomes. Ultimately, autophagosomes fuse with lysosomes to achieve the specific clearance of impaired mitochondria. This protective mechanism effectively suppress oxidative stress cascades at the source and apoptotic signals triggered by defective mitochondria, and consequently sustains cellular homeostasis (Wauer et al., 2015; Sukhorukov et al., 2021; Agarwal and Muqit, 2022).

Accumulating studies have further indicated that the PINK1/PARKIN cascade is not a linear signaling pathway but a sophisticated regulatory network governed by multiple positive and negative modulators (Thayer et al., 2026). PINK1-mediated phosphorylation of Mfn2 facilitates PARKIN recruitment and accelerates pathway activation. Additionally, TBK1 kinase phosphorylates autophagy receptors to enhance their substrate recognition and binding affinity, thereby optimizing mitophagic efficiency. In contrast, deubiquitinases including USP30 and USP15 counteract PARKIN-induced ubiquitination by erasing ubiquitin modifications from mitochondrial substrates, thereby exerting negative regulation on mitophagy. This negative feedback mechanism prevents the aberrant degradation of functionally intact mitochondria and maintains the dynamic homeostasis of mitochondrial quality control (Fritsch et al., 2020).

Distinct inhibitory mechanisms of USP30 and USP15 in gastrointestinal cells: USP30 is constitutively anchored to the mitochondrial outer membrane via its N-terminal hydrophobic domain, with catalytic domain exposed to cytosol; it preferentially erases K6/K11-linked ubiquitin chains on TOM20, VDAC1 and Mfn2, directly counteracting PARKIN-mediated ubiquitination on mitochondrial surface substrates, and reduces autophagy receptor recruitment without affecting PARKIN mitochondrial translocation. In contrast, USP15 distributes in the cytoplasm and only translocates to damaged mitochondria after PARKIN recruitment; it targets K48/K63 ubiquitin chains on both outer membrane substrates and activated PARKIN itself, indirectly attenuating PARKIN catalytic activity. In gastric smooth muscle and enterochromaffin cells of FD models, USP30 is persistently overexpressed under chronic oxidative stress to constitutively block mitophagy, while USP15 upregulation mainly occurs in late-stage mitochondrial injury to form secondary negative feedback inhibition. Multi-layered transcriptional and post-translational modulation jointly fine-tunes PINK1/PARKIN-mediated mitophagy. Transcription factors Nrf2, TFEB and SIRT1 directly upregulate PINK1 and PARKIN transcription to enhance cellular mitophagic capacity. Post-translational TBK1 phosphorylation optimizes autophagy receptor function, while USP30/USP15 counteract PARKIN activity to prevent overactive mitophagy. The dynamic balance between activating and inhibitory signals is critical for gastrointestinal mitochondrial homeostasis. Disruption of this balance may facilitate the progressive buildup progressive accumulation of damaged mitochondria, which may contribute to the initiation and progression of gastrointestinal disorders, particularly functional dyspepsia.

### 2.2 Core protective effects of the PINK1/PARKIN pathway on gastrointestinal cell functions

The PINK1/PARKIN pathway serves as a core signaling cascade for modulating mitochondrial quality homeostasis. By mediating selective mitophagy and preserving the structural and functional integrity of mitochondria, this pathway modulates the physiological activities of various functional cells in gastrointestinal tissues. Dysfunction of the PINK1/PARKIN pathway is closely implicated in the onset and progression of FD (Shangguan et al., 2017). Its core protective functions are summarized as follows:

#### 2.2.1 Sustaining gastrointestinal energy metabolism and normal motility

Mitochondria are the central organelles for cellular energy synthesis, providing sufficient ATP to support multiple physiological processes, including gastrointestinal smooth muscle contraction and epithelial substance transport (Haque et al., 2024). The PINK1/PARKIN pathway maintains the structural stability and efficient electron transport of the mitochondrial respiratory chain via timely clearance of damaged mitochondria, thereby ensuring the normal progression of aerobic respiration (Vincow et al., 2013). A preclinical animal study has demonstrated that inhibition of the PINK1/PARKIN pathway in gastric smooth muscle cells suppresses mitophagy and mitochondrial fission, which exacerbates gastric dysmotility in rats with spleen-deficiency-type FD. Accordingly, preclinical data suggest that dysfunction of this energy regulatory mechanism may serve as one contributor to FD-related motility defects (Zhang et al., 2022).

#### 2.2.2 Inhibiting oxidative stress injury and preserving gastrointestinal tissue integrity

Gastrointestinal mitochondria are major sites for reactive oxygen species (ROS) production. Damaged mitochondria tend to generate excessive ROS, triggering severe oxidative stress responses such as lipid peroxidation and protein oxidative modification. These aberrant alterations impair gastrointestinal mucosal epithelium and intestinal nerve terminals, thereby disrupting normal tissue function (Bhattacharyya et al., 2014). Multiple independent clinical and preclinical studies have validated the mucosal damaging effects of excess ROS in gastrointestinal tissues: sustained ROS accumulation disrupts tight junction protein expression to increase mucosal permeability, induces enteric nerve terminal oxidative apoptosis, and amplifies mucosal low-grade inflammatory responses (Chen et al., 2025c; Chen et al., 2025a; Chen et al., 2025b). Accumulating evidence has verified that oxidative stress-induced mitophagy is tightly associated with the PINK1/PARKIN signaling pathway (Garza-Lombó et al., 2020). This pathway selectively eliminates ROS-producing damaged mitochondria, blocks the initiation of oxidative stress at the source, and alleviates systemic oxidative damage. Such protective effects attenuate the destruction of the gastrointestinal mucosal barrier and nerve terminals, ultimately preserving the structural integrity and physiological sensory function of gastrointestinal tissues (Sulkshane et al., 2021).

## 3 Hypothesis and evidence chain linking the PINK1/PARKIN pathway with core pathological phenotypes of FD

FD is a prevalent functional gastrointestinal disorder with incompletely elucidated pathogenesis. Existing studies have confirmed that gastrointestinal dysmotility, visceral hypersensitivity, and chronic low-grade inflammation are the three core pathological phenotypes of FD (Ford et al., 2020), and abnormal mitophagy serves as a pivotal molecular basis underlying these pathological alterations (Qi et al., 2024). Recent clinical detection evidence has revealed significant mitochondrial structural damage and functional abnormalities in the gastric mucosa of FD patients, characterized by decreased mitochondrial membrane potential, accumulation of oxidative stress products (e.g., ROS and MDA), and insufficient ATP production. These findings indicate that the imbalance of mitochondrial quality control constitutes a crucial pathological foundation for FD occurrence.

Impaired mitophagy induced by PINK1/PARKIN pathway deficiency represents a tentative upstream molecular candidate implicated in FD pathogenesis based on animal and cellular evidence only. PINK1/PARKIN dysfunction leads to persistent accumulation of damaged mitochondria in gastric smooth muscle cells, intestinal epithelial cells and enterochromaffin cells, further triggering insufficient cellular energy supply, disordered oxidative stress, abnormal visceral sensory transmitter secretion and sustained low-grade mucosal inflammatory cascades. Collectively, these pathological changes induce the three core FD phenotypes and corresponding clinical manifestations, including postprandial fullness, early satiety, epigastric pain and burning sensation (**Figure 3**).

**Figure 3:**
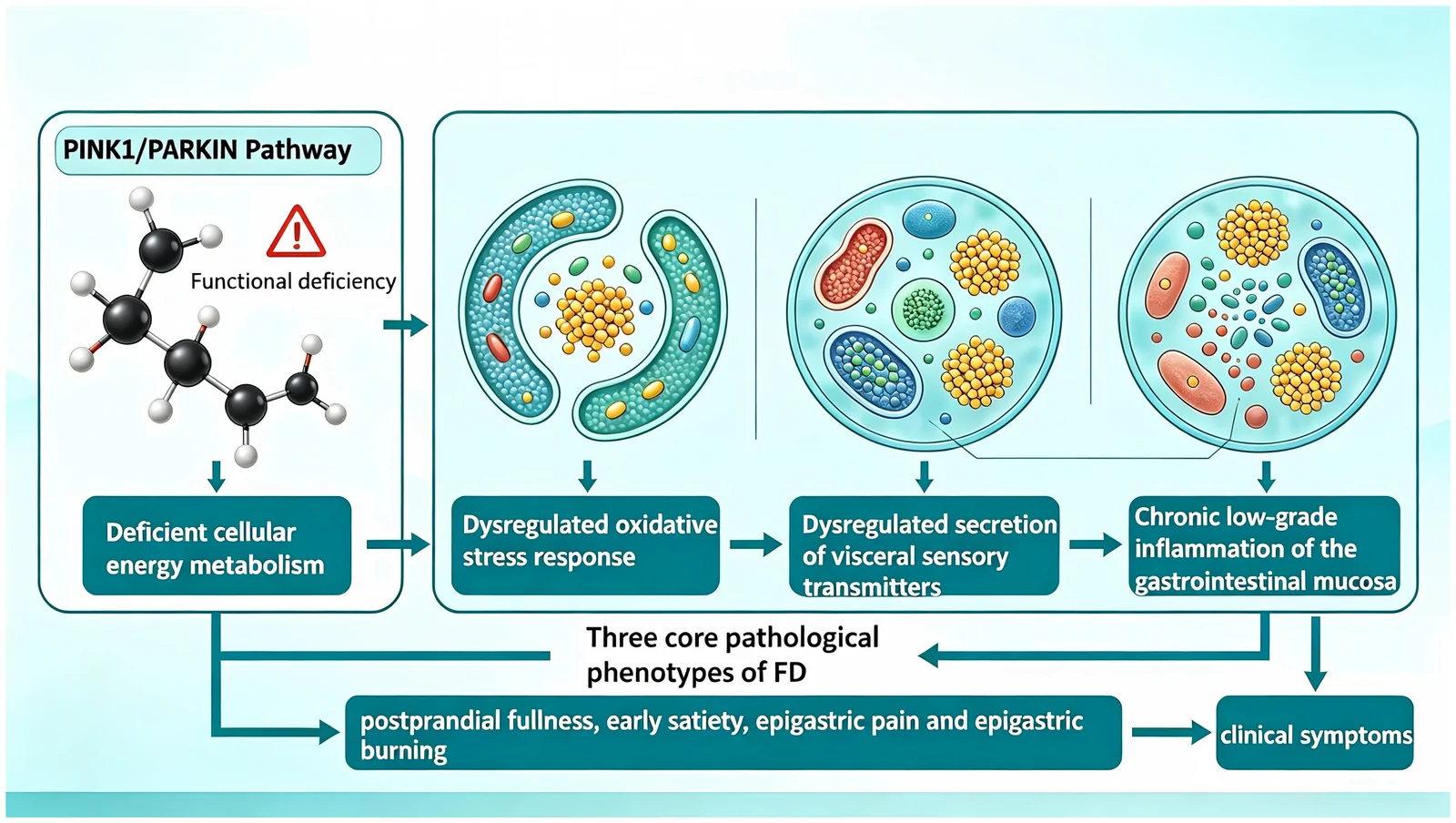
PINK1 PARKIN pathway dysfunction and the mechanism of FD pathogensis.

### 3.1 Gastrointestinal dysmotility (the predominant pathological phenotype of FD)

The rhythmic contraction and coordinated relaxation of gastrointestinal smooth muscles are prerequisite physiological processes for sustaining normal intestinal peristalsis, gastric emptying, and luminal food transportation. These energy-intensive biological behaviors rely predominantly on adenosine triphosphate (ATP) produced by mitochondrial respiratory chain oxidative phosphorylation. As the fundamental energy source for cellular physiological activities, sufficient ATP bioavailability is indispensable for maintaining normal gastric motor function. Impaired mitochondrial energy metabolism profoundly compromises smooth muscle contractility and rhythmic stability, thereby contributing to gastric hypomotility in FD pathogenesis (Lv et al., 2017; Laporte et al., 2020; Jin and Cai, 2021). Accumulating animal studies have demonstrated that reduced ATP content and overt mitochondrial structural damage are consistently observed in the gastric tissues of FD model rats compared with healthy counterparts, suggesting that defective mitophagy is a critical initiating factor for gastrointestinal motility dysfunction (Cui, 2023). Consistent with these findings, preclinical evidence further indicates that the expression levels of PINK1 and PARKIN are significantly suppressed in the gastric tissues of FD rats. Pharmacological activation of the PINK1/PARKIN-mediated mitophagy cascade effectively restores gastrointestinal motor function and ameliorates FD-like pathological phenotypes (Wang et al., 2024). Collectively, existing preclinical *in vivo* data only suggest a potential mechanistic correlation between impaired PINK1/PARKIN signaling and gastrointestinal hypomotility in FD animal models; robust human clinical verification remains lacking.

Based on the aforementioned evidence, this study constructs a rigorous and integrated molecular pathological cascade underlying FD pathogenesis. Specifically, functional impairment of the PINK1/PARKIN pathway suppresses selective mitophagic activity, resulting in progressive accumulation of damaged mitochondria within gastrointestinal smooth muscle cells. The retention of dysfunctional mitochondria disrupts the structural integrity of mitochondrial respiratory chain complexes and compromises electron transport efficiency, which substantially inhibits ATP biosynthesis and induces intracellular energy depletion. The resultant energy deficiency attenuates smooth muscle contractility and disrupts gastrointestinal electrophysiological rhythmicity, thereby delaying gastric emptying and suppressing intestinal peristalsis. These sequential pathological alterations ultimately contribute to the typical motility-dependent symptoms of FD, including postprandial fullness, early satiety, and upper abdominal distension.

### 3.2 Visceral hypersensitivity (core pain mechanism of FD)

Visceral hypersensitivity is a hallmark pathological abnormality of functional dyspepsia, characterized by the generation of adverse sensations in response to physiological stimuli and exaggerated perceptual sensitivity to noxious stimulation in visceral organs. Clinically, visceral hypersensitivity occurs in approximately 30%–50% of FD patients and is manifested as decreased sensory thresholds of the gastrointestinal tract to mechanical distension and chemical irritation, which represents a major contributor to abdominal pain symptoms in FD populations (Li and Shen, 2011; Rosen et al., 2014). Serotonin (5-hydroxytryptamine, 5-HT), predominantly secreted by gastrointestinal enterochromaffin cells, serves as a pivotal neurotransmitter regulating visceral sensory signal transduction. Upon specific binding to receptors on visceral afferent nerve terminals, 5-HT sensitizes nociceptive signaling pathways, reduces visceral pain thresholds, and augments gastrointestinal sensory responsiveness (Bayrer et al., 2023). Consistent with clinical observations, detectable upregulation of mucosal 5-HT abundance has been validated in gastric tissues from FD patients relative to healthy subjects, demonstrating that dysregulated synthesis and excessive release of 5-HT are critical molecular events underlying the initiation and maintenance of gastric visceral hypersensitivity.

The intracellular synthesis, vesicular trafficking, and extracellular secretion of 5-HT are tightly reliant on mitochondrial ATP production and precisely controlled by mitochondrial-dependent intracellular Ca homeostasis (Wang et al., 2012; Liu et al., 2021). In preclinical models, PARKIN loss-of-function may induce severe mitochondrial structural collapse and metabolic dysfunction within enterochromaffin cells, accompanied by excessive intracellular ROS accumulation and cytoplasmic calcium overload. These pathological alterations potentially overactivate 5-HT synthesis and promote vesicle exocytosis, which could lead to excessive peripheral release of 5-HT. Abnormally elevated 5-HT overstimulates peripheral visceral afferent nerves, amplifies pain signal transmission and reduces visceral sensory thresholds, which may facilitate the development of visceral hypersensitivity in FD (Stutzmann and Mattson, 2011; Gumeni et al., 2021; De-Miguel, 2022).

### 3.3 Vicious cycle of oxidative stress and low-grade inflammation

Chronic low-grade inflammation within the gastrointestinal mucosa is a key pathological driver of persistent symptoms and recurrent disease progression in FD (Doihara et al., 2009). Clinical evidence has validated markedly increased expression of pro-inflammatory cytokines, such as TNF-α and IL-6, in the gastric mucosa of FD patients relative to healthy individuals. The persistent inflammatory microenvironment further deteriorates gastrointestinal structural and functional integrity, aggravating FD pathological lesions (Singh et al., 2017). Within the inflammatory regulatory network, ROS functions as an essential upstream activator of the canonical NF-κB signaling cascade. ROS-driven phosphorylation cascades facilitate NF-κB nuclear translocation, which subsequently initiates the transcription, translation and secretion of downstream pro-inflammatory mediators, thereby sustaining and amplifying mucosal inflammatory responses.

Under physiological conditions, the PINK1/PARKIN cascade selectively clears damaged mitochondria to restrict ROS overproduction at its source. This inhibitory effect hinders NF-κB nuclear translocation and activation, interrupts downstream pro-inflammatory signaling, and sustains mucosal immune homeostasis within the gastrointestinal tract. In contrast, functional deficiency of the PINK1/PARKIN pathway results in aberrant accumulation of impaired mitochondria and persistent overproduction of ROS. Excess ROS may trigger oxidative damage to cellular lipids, proteins and DNA, and potentially sustain persistent NF-κB inflammatory signaling to facilitate the secretion of pro-inflammatory mediators such as TNF-α and IL-6. The chronic inflammatory microenvironment further exacerbates mitochondrial oxidative injury and aggravates insufficient mitophagy, ultimately forming a self-perpetuating vicious cycle of “mitochondrial damage-ROS accumulation-inflammatory activation-progressive mitochondrial dysfunction” (Kang, 2025). This pathological loop sustains unresolved gastrointestinal mucosal inflammation, contributes to intractable and recurrent FD symptoms, and leads to progressive deterioration of disease conditions.

## 4 Current research gaps and core scientific issues

Although accumulating preclinical and preliminary clinical evidence has suggested that aberrant mitophagy driven by PINK1/PARKIN pathway dysfunction is tightly implicated in FD pathogenesis, current studies remain largely preliminary and hypothesis-oriented. A comprehensive and systematic evidence chain is still lacking, with prominent research gaps in clinical relevance validation, subtype-specific pathological characteristics, and translational therapeutic exploration. Accordingly, in-depth investigations are urgently needed to clarify the underlying mechanisms and fill these critical research vacancies.

### 4.1 Three key research gaps

#### 4.1.1 Absence of direct clinical correlation evidence

To date, large-scale standardized clinical investigations characterizing changes to the PINK1/PARKIN cascade in FD patients are still absent. There is a lack of quantitative clinical data concerning the protein expression, kinase activity, and mitophagy activity of the PINK1/PARKIN pathway in human gastric mucosal tissues. Moreover, the quantitative associations between the magnitude of pathway dysfunction, disease phenotypes, and clinical symptom severity of FD remain uncharacterized. These unresolved issues preclude the definitive validation of the PINK1/PARKIN pathway as a core clinical molecular determinant governing FD initiation and progression.

#### 4.1.2 Unclarified mechanisms underlying FD subtype differences

Based on the Rome IV diagnostic criteria, functional dyspepsia is categorized into two predominant clinical subtypes, namely postprandial distress syndrome (PDS) and epigastric pain syndrome (EPS), which present distinguishable clinical symptoms and pathological features. However, whether discrepant alterations in PINK1/PARKIN pathway dysfunction, mitochondrial damage patterns, and mitophagic flux contribute to the phenotypic heterogeneity between PDS and EPS remains poorly elucidated. The absence of subtype-specific mechanistic evidence hinders the stratified molecular interpretation of FD pathogenesis and limits the in-depth understanding of disease heterogeneity.

#### 4.1.3 Unverified therapeutic efficacy of targeted intervention

At present, no studies based on FD cellular or animal models have validated the activating effects of specific PINK1/PARKIN pathway agonists or natural active compounds on this signaling cascade. Furthermore, whether such targeted interventions can reverse the core pathological phenotypes and clinical manifestations of FD remains unconfirmed. Collectively, the translational potential and clinical applicability of the PINK1/PARKIN pathway as a therapeutic target for FD lack sufficient experimental evidence and empirical verification.

### 4.2 Key scientific questions

Against the aforementioned research gaps and based on the scientific hypothesis proposed in this study, the core unresolved scientific questions are refined as follows, which provide clear theoretical directions for subsequent mechanistic and translational research: whether the protein expression and kinase activity of the PINK1/PARKIN pathway are significantly downregulated in the gastric mucosa of FD patients and exhibit quantitative correlations with disease severity; whether functional deficits in the PINK1/PARKIN pathway are the primary driver of defective mitophagy and abnormal accumulation of damaged mitochondria in gastrointestinal cells of FD patients; whether targeted activation of the PINK1/PARKIN pathway could effectively rescue the three core pathological hallmarks of FD, including gastrointestinal motility dysfunction, visceral hypersensitivity, and chronic low-grade inflammatory injury; and whether key molecules associated with the PINK1/PARKIN pathway can be exploited as novel non-invasive biomarkers for FD clinical diagnosis, subtype discrimination, and therapeutic response assessment.

## 5 Future research directions and translational prospects

Addressing the aforementioned research deficiencies and core scientific controversies, this chapter systematically proposes prospective research directions covering clinical correlation verification, mechanistic exploration, and translational development, in accordance with the latest advances in mitochondrial quality control and functional gastrointestinal disorder research. This framework aims to elaborate the theoretical significance and clinical translational potential of the PINK1/PARKIN pathway in the pathological progression, diagnosis, and targeted intervention of FD.

### 5.1 Clinical research: validating the intrinsic correlation between the PINK1/PARKIN pathway and FD

A multicenter, large-scale cross-sectional clinical study will be implemented in strict compliance with the Rome IV diagnostic criteria. FD patients with PDS and EPS subtypes will be enrolled, and age- and gender-matched healthy subjects will be recruited as the control cohort. Gastric mucosal biopsy specimens will be harvested from all participants for quantitative detection of core mitophagy-related molecules, including PINK1, PARKIN, p62, and the LC3-II/LC3-I ratio, alongside comprehensive assessments of mitochondrial function and oxidative stress profiles. Combined with objective evaluations of gastric emptying function and visceral sensory sensitivity, correlation analyses will be conducted to delineate the quantitative relationships between PINK1/PARKIN pathway dysfunction, disease phenotypic characteristics, and clinical symptom severity. These efforts will help consolidate the core clinical status of dysregulated PINK1/PARKIN-mediated mitophagy in FD pathogenesis.

### 5.2 Basic research: elucidating the causal mechanisms underlying PINK1/PARKIN pathway-mediated FD regulation

Physiologically relevant cellular and animal models that faithfully recapitulate FD pathophysiological features will be established for mechanistic validation. *In vitro*, lipopolysaccharide-stimulated injury models of gastrointestinal smooth muscle cells and enterochromaffin cells will be utilized. *In vivo*, chronic restraint stress and water-immersion restraint stress paradigms will be employed to construct FD experimental animal models. Genetic modulation strategies, including gene knockdown, overexpression, and gene editing, will be applied to precisely manipulate the expression and functional activity of the PINK1/PARKIN pathway. A comprehensive panel of observational indicators will be systematically measured, encompassing mitochondrial structural and functional integrity, mitophagic flux dynamics, energy metabolic status, oxidative stress levels, 5-HT secretion efficiency, pro-inflammatory cytokine expression, smooth muscle contractile activity, gastric emptying rate, and visceral pain threshold. These multidimensional detections are intended to clarify the definite causal relationship and underlying molecular mechanisms by which the PINK1/PARKIN pathway governs FD pathological progression.

### 5.3 Translational research: targeted intervention and biomarker development

Traditional Chinese herbal therapies show promising therapeutic potential for FD management. A growing body of preclinical evidence suggests that classic TCM prescriptions may relieve FD-related pathological phenotypes by activating PINK1/PARKIN-dependent mitophagy and recovering mitochondrial quality control homeostasis. These TCM interventions significantly upregulate PINK1 and PARKIN expression, promote PARKIN mitochondrial translocation, increase LC3-II/LC3-I ratio and reduce p62 accumulation, accelerating clearance of damaged mitochondria, reducing ROS overproduction, recovering ATP supply, improving gastric smooth muscle contraction, attenuating visceral hypersensitivity and suppressing chronic mucosal inflammation.

Comparative translational profiling of three natural phytochemicals as PINK1/PARKIN-targeted agents for FD intervention. Resveratrol, curcumin and berberine represent well-documented botanical bioactive compounds capable of restoring defective PINK1/PARKIN-mediated mitophagy, yet they exhibit distinct pharmacological merits and translational bottlenecks when applied for FD management.

#### 5.3.1 Resveratrol

Strengths: It may exert broad-spectrum mitochondrial protective effects by concurrently stimulating the upstream SIRT1/Nrf2 transcriptional axis to augment PINK1/PARKIN signaling. Additionally, resveratrol displays acceptable oral gastrointestinal bioavailability, and multiple mature pharmaceutical delivery systems have been established to facilitate its clinical translation.

Limitations: The compound possesses a short circulating half-life after oral intake, resulting in unsatisfactory systemic exposure. Moreover, chronic administration at high doses may trigger hyperactive mitophagy, which potentially disrupts physiological mitochondrial turnover and impairs normal cellular homeostasis.

#### 5.3.2 Curcumin

Strengths: Curcumin potentially produces potent anti-inflammatory activity via direct transcriptional upregulation of PINK1, and it carries minimal systemic toxic risk, rendering it preferential for FD patients dominated by chronic low-grade mucosal inflammation.

Limitations: Its clinical utility is hampered by intrinsic poor aqueous solubility and inefficient intestinal absorption, which necessitates nanocarrier modification to enhance bioavailability. Furthermore, curcumin fails to sufficiently promote PARKIN mitochondrial translocation, restricting its capacity to fully reactivate mitophagic flux.

#### 5.3.3 Berberine

Strengths: Berberine preferentially accumulates within gastric mucosal tissues following oral administration, enabling dual modulation of intestinal microecology and PINK1/PARKIN-dependent mitophagy. This unique dual property makes it a promising candidate for FD patients complicated with gut dysbiosis.

Limitations: Berberine barely distributes into peripheral circulating tissues, limiting its systemic mitochondrial regulatory effects. Long-term oral supplementation may perturb the balance of commensal intestinal flora and trigger adverse gastrointestinal reactions such as dry mouth and constipation.

Targeted activation of the PINK1/PARKIN mitophagy pathway represents a promising potential therapeutic strategy for FD prevention and treatment. Multiple candidate intervention reagents have been identified, including natural small-molecule compounds, autophagy agonists (rapamycin, metformin), mitochondrial-targeted antioxidants (MitoQ) and USP30 specific inhibitors. These interventions enhance PINK1 kinase activity, facilitate PARKIN mitochondrial recruitment, promote mitochondrial protein ubiquitination and rescue defective mitophagic flux, thereby reversing the core pathological abnormalities of FD.

For translational biomarker development, peripheral blood indicators including circulating monocyte/platelet PINK1/PARKIN mRNA levels and mtDNA copy number possess potential value as non-invasive biomarkers for auxiliary FD diagnosis, subtype classification and dynamic therapeutic response monitoring, avoiding the invasiveness of gastroscopic biopsy and optimizing clinical screening workflows.

Among peripheral blood cells, circulating monocytes show far more stable mitophagy-related molecular signals for clinical detection than platelets. Monocytes contain complete intact mitochondria with full PINK1/PARKIN transcriptional machinery; their mtDNA, PINK1/PARKIN mRNA levels are linearly correlated with gastric mucosal mitophagy damage with low inter-individual variation. By contrast, platelets are anucleate cell fragments with scarce mitochondria; their mitophagy markers are easily disturbed by inflammation and platelet activation, leading to large detection deviation in clinical cohorts (Wauer et al., 2015; Fritsch et al., 2020).

## 6 Conclusion

The PINK1/PARKIN pathway acts as a core signaling axis modulating mitophagy and mitochondrial homeostasis in gastrointestinal cells, exerting multiple protective biological effects including sustaining cellular energy metabolism, alleviating oxidative stress injury, regulating visceral sensory signal transduction, and preserving the integrity of the gastrointestinal mucosal barrier. Parallel to the PINK1/PARKIN cascade, the non-ubiquitin BNIP3/NIX mitophagy axis also participates in gastrointestinal mitochondrial homeostatic regulation under the hypoxic and inflammatory microenvironment associated with FD, though its specific pathological mechanisms remain insufficiently elucidated.Based on currently available preclinical experimental data, we tentatively hypothesize that impaired mitophagy induced by PINK1/PARKIN pathway suppression may represent one candidate upstream molecular factor contributing to FD initiation and progression. Dysregulated PINK1/PARKIN-mediated mitophagy is hypothesized to drive three core pathological phenotypes of FD: gastrointestinal motility dysfunction, visceral hypersensitivity, and chronic low-grade mucosal inflammation.

Nevertheless, existing investigations within this field remain preliminary and hypothesis-driven, lacking sufficient direct human clinical evidence and rigorous causal verification of molecular mechanisms, and a comprehensive, systematic research framework has not yet been established. Future research focusing on clinical correlation validation, mechanistic dissection, targeted drug screening and biomarker discovery is required to consolidate the evidence chain linking dysfunctional PINK1/PARKIN-dependent mitophagy to FD pathogenesis. Such follow-up work may offer novel mechanistic interpretations, precise therapeutic targets and non-invasive diagnostic strategies for FD. Incorporating the PINK1/PARKIN regulatory axis into FD research realizes interdisciplinary integration between mitochondrial quality control research and functional gastrointestinal disorder studies. This innovative research perspective carries important academic significance and promising clinical translational potential, pending large-scale human trials for definitive confirmation.

## Data Availability

The original contributions presented in the study are included in the article/Supplementary Material. Further inquiries can be directed to the corresponding authors.
